# Inflammation and kidney injury attenuated by prior intake of Brazil nuts in the process of ischemia and reperfusion

**DOI:** 10.1590/2175-8239-JBN-2018-0016

**Published:** 2018-08-16

**Authors:** Maria Fernanda Ribeiro Cury, Estéfany Queiroz Olivares, Renata Correia Garcias, Giovana Queda Toledo, Natassia Alberici Anselmo, Leticia Colombo Paskakulis, Fernanda Fortuci Resende Botelho, Natiele Zanardo Carvalho, Analice Andreoli da Silva, Camila Agren, Carla Patrícia Carlos

**Affiliations:** 1 Laboratório de Pesquisa Experimental, FACERES Faculdade de Medicina, São José do Rio Preto, SP, Brasil.

**Keywords:** Ischemia, Reperfusion, Acute Kidney Injury, Inflammation, Bertholletia, Rats, Isquemia, Reperfusão, Lesão Renal Aguda, Inflamação, Bertholletia, Ratos

## Abstract

**Introduction:**

Ischemia and reperfusion (IR) is a process inherent to the procedures involved in the transplantation of organs that causes inflammation, cell death and cell injury, and may lead to rejection of the graft. It is possible that the anti-inflammatory properties of the Brazil nuts (BN) can mitigate the renal injury caused by IR.

**Objective:**

To investigate whether the previous intake of BN reduces the expression of markers of inflammation, injury, and cell death after renal IR.

**Methods:**

Male Wistar rats were distributed into six groups (N = 6/group): SHAM (control), SHAM treated with 75 or 150 mg of BN, IR, and IR treated with 75 or 150 mg of BN. The IR procedure consisted of right nephrectomy and occlusion of the left renal artery with a non-traumatic vascular clamp for 30 min. BN was given daily from day 1 to 7 before surgery (SHAM or IR), and maintained until sacrifice (48 h after surgery). Inflammation was evaluated by renal expression of COX-2 and TGF-β, injury by the expression of vimentin, and cell death by apoptosis through caspase-3 expression (immunohistochemistry).

**Results:**

Pretreatment with 75 mg of BN reduced renal expression of the COX-2, TGF-β, vimentin, and caspase-3. The dose of 150 mg caused increased expression of COX-2.

**Conclusion:**

In experimental IR, the damage can be minimized with a prior low-dose intake of BN, improving inflammation, injury, and cell death.

## RESUMO

**Introdução:**

Isquemia e reperfusão (IR) é um processo inerente aos procedimentos envolvidos no transplante de órgãos, que causa inflamação, morte celular e lesão, podendo levar à rejeição do enxerto. É possível que a castanha-do-brasil (CB), por suas propriedades anti-inflamatórias, possa atenuar a lesão renal causada pela IR.

**Objetivo:**

Investigar se a ingestão prévia de CB reduz a expressão de marcadores renais de inflamação, lesão e morte celular após a IR.

**Métodos:**

Ratos Wistar machos foram distribuídos em seis grupos (N = 6/grupo): SHAM (controle), SHAM tratado com 75 ou 150 mg de CB, IR, e IR tratado com 75 ou 150 mg de CB. O procedimento de IR consistiu na nefrectomia à direita e oclusão da artéria renal esquerda por 30 minutos. A castanha foi administrada diariamente por sete dias antes da cirurgia (SHAM ou IR), e mantida até o sacrifício (48 horas pós-cirurgia). A inflamação foi avaliada pela expressão renal de COX-2 e TGF-β; a lesão pela expressão de vimentina, e a morte celular por apoptose pela expressão de caspase-3, por imuno-histoquímica.

**Resultados:**

O pré-tratamento com 75 mg de CB reduziu a expressão renal de COX-2, de TGF-β, de vimentina e de caspase-3. A dose de 150 mg causou elevação da expressão de COX-2.

**Conclusão:**

No modelo experimental de IR renal, os danos podem ser minimizados com a ingestão prévia de baixas doses de CB, melhorando a inflamação, a lesão e a morte celular.

## INTRODUCTION

Ischemia and reperfusion (IR) is a major cause of acute renal failure and graft rejection. This condition occurs during the procedures involved in the transplantation of organs, and the damages in kidney are associated with reactive oxygen species produced after blood reperfusion. A cascade of cellular responses leads to inflammation, cell death and organ failure.^1^^-^ 4 Therefore, the understanding of the mechanisms involved in this injury is essential to minimize the consequences of the procedures involved in renal transplantation. Thus, our study proposed an investigation of the protective action of Brazil nut (BN), *Bertholettia excelsa*, in inflammation and cell death caused by acute renal injury during experimental IR in rats.

It is known that the regular consumption of BN improves the lipid profile and microvascular function, and reduces oxidative stress in obese teens,^5^ an effect also observed in subjects with metabolic syndrome,^6^ reducing the atherogenic risk in obese women, with increased activity of glutathione-peroxidase.^7^ Healthy volunteers taking BN present reduced inflammation markers, such as IL-1 (interleukin 1), IL-6, TNF-α (tumor necrosis factor alpha), and IFN-γ (gamma interferon),^8^ and improved lipid profile for a period exceeding 30 days.^9^ Thus, it is clear that BN has a protective effect in diseases related to oxidative stress and inflammation. Also, this benefit may be related to its bioactive compounds selenium, tocopherol, phenolic compounds, folate, magnesium, and mono/polyunsaturated fatty acids.^10^^-^ 11

Given the increased risk of mortality due to acute renal failure and the fact that the anti-inflammatory activity of BN have been poorly explored in IR-induced kidney injury, the aim of the present study was to investigate whether the previous intake of this nut reduces the expression of the markers of inflammation, injury, and cell death after renal IR.

## METHODS

### ETHICS

All procedures performed in this study were in accordance with the ethical standards approved by the Committee for Animal Experiments and the Ethics Committee of FACERES School of Medicine (approval number 001/2015).

### ANIMALS AND PROCEDURES

Male Wistar rats (200-220 g) were randomly distributed into six groups (N = 6/group): SHAM (control), SHAM treated with 75 or 150 mg of BN (SHAM+BN), IR, and IR treated with 75 or 150 mg of BN (IR+BN). The animals were housed under a 12:12 h light-dark cycle and allowed access to food and water ad libitum.

The IR procedure consisted of right nephrectomy and occlusion of the left renal artery with a non-traumatic vascular clamp for 30 min under anesthesia (xylazine 10 mg/Kg + ketamine 85 mg/Kg).^12^^-^ 14

BN (75 or 150 mg/animal, Belém do Pará, Brazil) were given daily and individually from day 1 to 7 before surgery (SHAM or IR) and maintained until animal sacrifice (48 h after surgery). The doses were selected according to previous studies of humans who ingested nuts in doses without nephrotoxic and hepatotoxic effects.^5^^-^ 9 The amount of BN was adjusted daily according to the weight of the animal. Each rat was allocated to a single cage, and BN was offered separate from the feed. As it is very palatable, the nut had no rejection and was consumed almost immediately by the animals, even in the postoperative period.

Animals from the SHAM groups were submitted to the same anesthesia and surgical procedures described above but without the renal artery clamped. The animals were euthanized 48 h after reperfusion under overdose of anesthetic.

At the end of the surgery, all the animals were given tramadol 2 mg/Kg by gavage for postoperative pain control, kept in individual cages, and received food and water ad libitum for 48 hours. Nut intake was kept, according to the group, until the moment of sacrifice.

### KIDNEY MARKERS OF INFLAMMATION, INJURY, AND CELL DEATH

After 2 days of IR or SHAM procedure, the rats were euthanized by an overdose of the anesthetic (thiopental 100 mg/kg) and renal tissue samples were collected, fixed in a 4% paraformaldehyde in PBS 0.1 M (pH 7.4) for 24 h at 4°C, and embedded in paraffin. The study was performed by immunohistochemistry method as described before^15^^-^ 16 with immunoperoxidase reaction. The tissue fragments were incubated overnight at 4°C with primary antibodies. The inflammation was studied using anti-COX-2 (1:500, ab62331, ABCAM, Cambridge, UK) and anti-TGF-β (1:80, SC-7892 policlonal, Santa Cruz, CA, USA) markers. The markers for apoptosis and injury were anti-caspase-3 (1:1000, 9662, Cell Signaling Technology, Danvers, MA, USA) and anti-vimentin (1:500, M0725, Dako, Denmark). Twenty-five fields of the renal juxtamedullary region were evaluated in one slide from each animal to obtain an average of the scores performed as previously described.^14^^-^ 16

### STATISTICAL ANALYSIS

The data were previously subjected to descriptive analysis and determination of normality using the Kolmogorov-Smirnov test. We applied analysis of variance (ANOVA), followed by the Tukey's post-hoc test for multiple comparisons of samples with a normal distribution. The Kruskal-Wallis test followed by Dunn's test was used for samples with a non-normal distribution. A *p* value ≤ 0.05 was considered significant.

## RESULTS

### BRAZIL NUT REDUCES THE EXPRESSION OF MARKERS OF APOPTOSIS AND RENAL INJURY INDUCED BY IR

**Figure 1:**
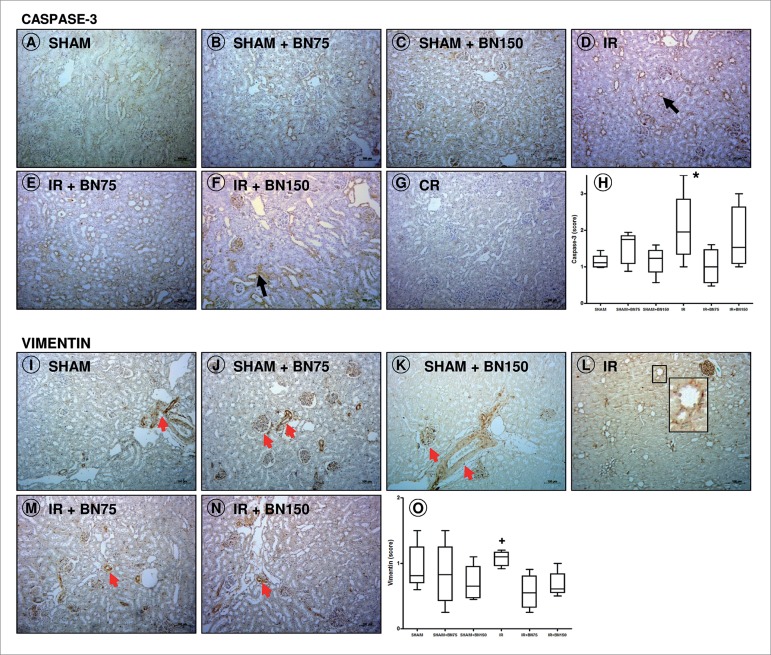
Injury and cell death in renal tissue after ischemia and reperfusion (IR) and pretreatment with Brazil nuts (BN): expression of caspase-3 (A-H) and vimentin (I-O). IR group shows increased expression of caspase-3 (black arrows, D/H) and vimentin (L/O) in the tubules and interstitium. The pretreatment with 75 mg (M/O) and 150 mg (N/O) reduced the cellular injury. [L] Detail of vimentin expression in the IR group. Red arrows indicate vimentin expression normally displayed in the blood vessels and glomerulus. Cell death was also mitigated by the BN dose of 75 mg (E/H), but not with the 150 mg dose (F/H). [G] Control reaction (CR). [H/O] Average score. Counterstain: Hematoxylin. Bars: 100 µm. *p<0.05, IR *vs*. IR+BN75; ^+^ *p*<0.05 IR *vs*. IR+BN150 (ANOVA + Tukey's post-test). Data presented as medians, quartiles 25-75%, minimum and maximum values.

The animals of the IR group exhibited increased expression of caspase-3 (Figure 1 D/H) compared with IR+BN75 (Fig.1 E/H). A similar result was observed for the expression of vimentin (Fig. 1 L/O), compared to IR groups treated with 75 (Fig. 1 M/O) or 150 mg of BN (Fig. 1 N/O).

### PRIOR LOW DOSE INTAKE OF BN REDUCES THE EXPRESSION OF THE TGF-β AND HIGH DOSE INCREASES COX-2

**Figure 2:**
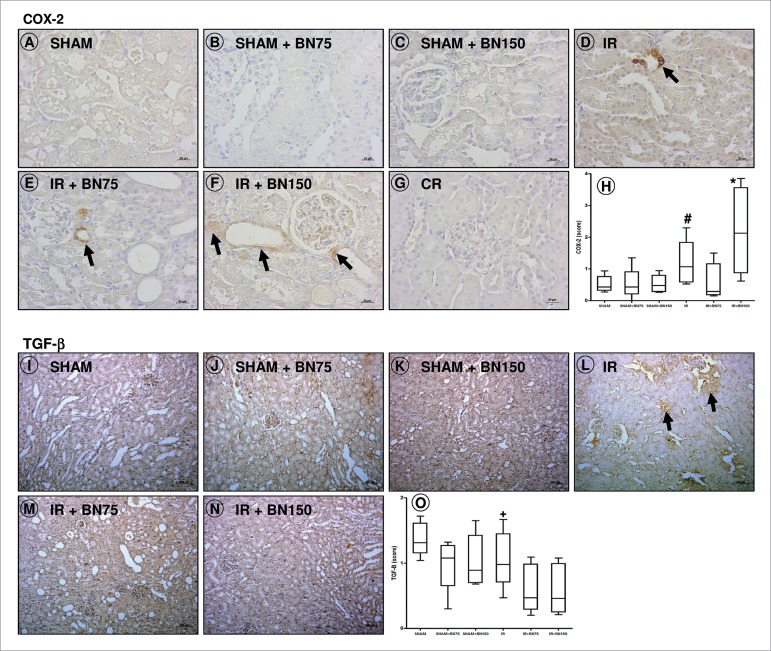
Inflammation in the renal tissue after ischemia and reperfusion (IR) and pretreatment with Brazil nuts (BN): expression of COX-2 (A-H) and TGF-β (I-O). Treatment with 150 mg of BN (F/H) 7 days before IR surgery increased the expression of cyclooxygenase-2 (COX-2) compared to other groups. The IR group had increased expression of COX-2 (D/H) and TGF-β (L/O), but treatment with 75 mg (E/H and M/O) mitigated this effect. [G] Control reaction (CR). [H/O] Average score. Counterstain: Hematoxylin. Bars: 100 µm. ^#^ *p*<0.05, IR vs. SHAM, and *p<0.05, IR+BN150 *vs*. all groups (Kruskal-Wallis + Dunn's post-test); ^+^ *p*<0.05 IR *vs*. IR+BN75 and IR+BN150 (ANOVA + Tukey's post-test). Data presented as medians, quartiles 25-75%, minimum and maximum values.

The IR groups presented elevation in COX-2 (Figure 2 D/H)) compared with SHAM group (Fig. 2 A/H); this marker was higher in the IR group treated with 150 mg of BN (Fig. 2 F/H) compared with all groups. The expression of TGF-β was higher in the IR group (Fig. 2 L/O) compared to IR groups treated with 75 (Fig. 2 M/O) or 150 mg of BN (Fig. 2 N/O).

## DISCUSSION

IR-induced renal injury causes the release of reactive oxygen species (ROS), proinflammatory mediators, and adhesion molecules, and leukocyte recruitment; together, these processes induce kidney dysfunction and mortality.^1^^,^ 4 ^,^ 17 ^-^ 18 In a previous study, we verified that treatment with 75 mg of BN seven days before the IR process attenuated the deleterious effects of IR on renal function, such as reduction of plasmatic urea and proteinuria, and increased creatinine clearance and urine volume. These improvements are related to the inhibition of macrophage infiltration and oxidative stress. However, kidney injury was not different among the IR groups treated or not with 75 mg and 150 mg of BN.^14^ Thus, in the present study, we tested whether the intake of this nut reduces the expression of the markers of kidney inflammation, injury, and cell death after renal IR.

We studied the expression of vimentin in juxtamedullary region of IR kidney rats. Under physiologic conditions, renal vimentin is found in the arterial smooth muscle cells and in the glomerulus, but not in the tubular cells.^15^^-^ 16 ^,^ 19 Kidney injury induces changes in the vimentin expression. Renal epithelial tubular cells may modify their phenotype, adopting characteristics of mesenquimal cells such as fibroblasts, which are involved in extracellular matrix deposition and fibrosis development. This process is called epithelial-mesenchymal transition, or EMT,^20^^-^ 21 and is mediated by TGF-β1.^20^^-^ 23 EMT has been described in IR, and may contribute to the genesis of late fibrosis observed in this condition.^24^^-^ 27 Vimentin is useful as marker of injury and EMT development, and change in its expression pattern indicates proximal tubular injury.^15^^-^ 16 ^,^ 19 ^,^ 28 In our study, consistent with previously published data,^24^^-^ 27 ^,^ 29 ^-^ 30 IR-induced enhancement of vimentin expression in the renal tubule and interstitium in the juxtamedullary region was observed, suggesting proximal tubule cell damage. Both treatments, 75 and 150 mg of BN seven days before the IR procedure, reduced the tubular injury with lower expression of vimentin. The changes attenuated by BN corroborate the protective role of the nut observed previously.^14^

As reported before, IR condition causes elevation of kidney macrophage influx.^14^ Here, we verified that IR caused elevation in COX-2 expression, confirming kidney inflammation. One of the cytokines released by the macrophages is the TGF-β,^31^ and its release may be induced by the IR process,^24^^,^ 26 ^,^ 30 as was confirmed in this study. TGF-β is largely involved in the development of interstitial fibrosis and progression of chronic kidney disease.^24^^,^ 26 ^,^ 31 ^-^ 32 As reported, it is related to the EMT process.^20^^-^ 23 Kidney macrophage influx^14^ and TGF-β expression after IR procedure are reduced by previous treatment with both BN doses of 75 and 150 mg. These results are corroborated by the anti-inflammatory action demonstrated by studies of BN consumption by healthy humans^8^ and by renal disease patients^33^^-^ 35, and indicate that low intake of BN is a promising alternative during IR procedure. It would be interesting to verify its effect on the progression of chronic kidney disease. However, the dosage of 150 mg of BN elevated the kidney COX-2 expression after IR, which confirms the findings reported earlier that the highest dose of BN is harmful to the IR procedure.^14^

In addition to the inflammatory mediators, we studied the kidney cell death by apoptosis after IR and BN treatment by checking the expression of caspase-3. The kidneys of the IR group presented elevation of apoptosis in accordance with other authors.^4^^,^ 36 ^-^ 37 The IR process induces nitric oxide expression in tubule cells generating ROS^14^^,^ 36, which cause renal tubule cell injury by oxidation of proteins, peroxidation of lipids, damage to DNA, and induction of apoptosis.^1^^,^ 4 ^,^ 36 As proposed by Devarajan,^36^ there is evidence that apoptosis is the main mechanism of early tubule cell death in contemporary clinical acute renal failure, and the inhibition of apoptosis and inflammation at this stage may represent a powerful therapeutic approach. In fact, our study demonstrates that the previous treatment with 75 mg of BN reduces the caspase-3 expression in kidneys after IR, confirming its protective effect against cell death.

Thus, our study shows that BN exerts a beneficial effect on renal injury and inflammation and improves the function harmed by the IR as demonstrated previously.^14^

However, unlike treatment with 75 mg of BN, the kidney COX-2 expression was higher in the BN150 group. Corroborating these data, the kidney function of IR animals got worse with the intake of 150 mg of BN. The plasmatic creatinine, urea, and phosphorus were elevated compared to the other groups.^14^ As explained, this effect may be related to elevated phosphorus and amino acids content of the BN.^11^ Electrolytes disorders are commonly encountered in kidney diseases, and nutritional support is frequently needed^38^ such as the low intake of protein.^39^^-^ 40 Therefore, a higher consumption of nuts could harm this nutritional management. In fact, the ingestion of only one Brazil nut per day is recommended for hemodialysis patients to get the anti-inflammatory and antioxidant protective effect.^33^ Our study reinforces this daily maximum recommendation of nuts for patients with kidney diseases. Moreover, it indicates a possible pro-inflammatory effect of 150 mg of BN. Thus, the mechanism involved in this pro-inflammatory effect needs to be better investigated. In addition, it would be interesting to verify the effect of BN in chronic kidney disease to address the protective effect on renal injury caused by ischemia and reperfusion.

## CONCLUSION

In summary, the results point to a beneficial effect of a prior intake of a low dose of BN on kidney damage caused by the IR procedure, improving inflammation, injury, and cell death.
